# Early-life exposure to the Chinese famine of 1959–61 and risk of Hyperuricemia: results from the China health and retirement longitudinal study

**DOI:** 10.1186/s12889-019-8017-1

**Published:** 2020-01-06

**Authors:** Wenqiang Zhang, Rongsheng Luan

**Affiliations:** West China School of Public Health and West China Fourth Hospital, Sichuan University, No. 16, Section 3, Ren Min Nan Road, Chengdu, 610041 Sichuan China

**Keywords:** Starvation, Hyperuricemia, Prenatal exposure delayed effects

## Abstract

**Background:**

Short-term starvation has been related to hyperuricemia. However, little is known about the long-term effect of early-life exposure to famine on hyperuricemia risk in adulthood.

**Methods:**

The analysis included 2383 participants from the China Health and Retirement Longitudinal Study in 2015. Hyperuricemia was diagnosed as serum uric acid ≥7 mg/dL in men and serum uric acid ≥6 mg/dL in women. Famine exposure subgroups were defined as unexposed (born between October 1, 1962, and September 30, 1964), fetal-exposed (born between October 1, 1959, and September 30, 1961), and early-childhood exposed (born between October 1, 1956, and September 1, 1958). The association between early-life famine exposure and hyperuricemia risk was assessed using multivariate logistic regression.

**Results:**

The prevalence of hyperuricemia in the unexposed, fetal-exposed, and early-childhood exposed participants was 10.7, 14.1, 11.1%, respectively. Compared with unexposed and early-childhood exposed participants combined as an age-balanced control, fetal-exposed participants showed an increased risk of hyperuricemia in adulthood (*OR* = 1.41; 95% *CI*: 1.06–1.88), after adjusting for gender, marital status, famine severity, residence, smoking, drinking, BMI, hypertension, and diabetes. The famine effect on hyperuricemia was accentuated by overweight or obesity (*P* for interaction = 0.042). Compared with unexposed and BMI < 24 kg/m^2^ participants, the *OR* (95%*CI*) of hyperuricemia was 3.66 (2.13–6.30) for fetal-exposed and overweight/obesity participants. However, combined unexposed and early-childhood exposed participants as an age-balanced control, the interaction of famine exposure and BMI was not statistically significant (*P* for interaction = 0.054).

**Conclusion:**

Famine exposure in the fetal stage was associated with an increased risk of hyperuricemia in adulthood.

## Background

Serum uric acid (SUA) is a natural product of purine metabolism. The overproduction or underexcretion of uric acid can result in hyperuricemia [1, 2]. Recently, the prevalence of hyperuricemia has been increasing throughout the world [3–5]. A national cross-sectional survey in Chinese adults showed the prevalence of hyperuricemia was 8.4%, approximately 92.9 million adults with hyperuricemia [6]. Furthermore, hyperuricemia is the dominant risk factor for gout and might cause worse outcomes in cardiovascular disease and chronic kidney disease [7–9].

Both non-genetic and inherited genetic risk factors can contribute to hyperuricemia [2]. Previous epidemiological studies indicated hyperuricemia was associated with age, gender, obesity, drinking, smoking, dietary, genes [2, 3, 10–14]. In clinical studies, short-term starvation has been related to hyperuricemia [15–17]. However, epidemiological evidence for the long-term effect of early-life famine exposure on hyperuricemia in later life was still limited.

The Chinese Great Famine in 1959–61 lasted for a relatively prolonged period, which could provide us with a natural experiment to investigate the association between famine in early life and hyperuricemia risk. In the present study, we used the data from the China Health and Retirement Longitudinal Study in 2015 to examine whether early-life exposure to the Chinese famine of 1959–61 increased the risk of hyperuricemia in later life.

## Methods

### Participants

**Fig. 1:**
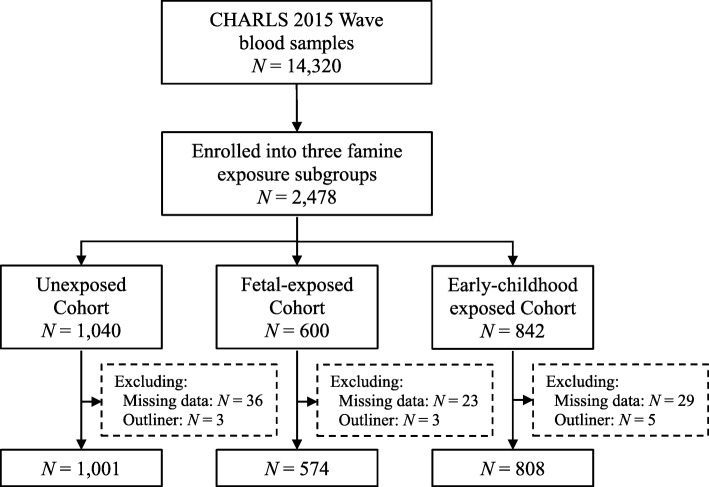
Flowchart on the sample selection and exclusion

The China Health and Retirement Longitudinal Study (CHARLS) is a nationally representative longitudinal survey of persons aged 45 years or older and their spouses, including household questionnaire, clinical measurements, and blood biomarkers [18]. For the current study, 2482 participants were enrolled into three famine exposure subgroups from 14,320 venous blood samples in the 2015 Wave of CHARLS. We dropped 99 participants who had missing data or outliners (Additional file 1: Table S1). As a result, the analysis included 2383 participants (Fig. 1).

### Assessment of famine exposure and severity

We categorized participants by actual birthdate into three famine exposure subgroups:

unexposed (born between October 1, 1962, and September 30, 1964), fetal-exposed (born between October 1, 1959, and September 30, 1961), and early-childhood exposed (born between October 1, 1956, and September 1, 1958). To avoid the age-related biases [19], we combined unexposed and early-childhood exposed subgroup as an age-balanced control.

The severity of Chinese famine varied remarkably across regions and time [19]. As shown in previous studies [20–23], provincial excess mortality in 1956–62 was computed as an indicator of famine severity, and excess mortality of 50% was used to classify provinces into severely affected and less severely affected areas.

### Diagnosis of hyperuricemia

As widely accepted in previous studies [6, 24–26], hyperuricemia was defined as SUA ≥ 7 mg/dL for men or SUA ≥ 6 mg/dL for women.

### Covariates

Hypertension was defined as a self-report of doctor diagnosis or systolic blood pressure ≥ 140 mmHg or diastolic blood pressure ≥ 90 mmHg. Diabetes was defined as a self-report of doctor diagnosis or fasting blood glucose ≥126 mg/dL or HbA1c ≥ 6.5%. Gender (male = 1 and female = 0), marital status (if married and living with spouse = 1, and 0 otherwise), smoking (former or current = 1, and never = 0), drinking (former or current = 1, and never = 0), residence (urban = 1 and rural = 0), hypertension (yes = 1 and no = 0), diabetes (yes = 1 and no = 0) were coded as dichotomous variables. The body mass index (BMI) was calculated as weight in kilograms divided into height in meters squared (kg/m^2^). Based on the criteria recommendation for Chinese adult [27], overweight or obesity were defined as BMI ≥ 24.0 kg/m^2^.

### Statistical analysis

Pearson’s *Chi*-Square test was used to detect the difference of categorical variables between two famine-exposed subgroups and the unexposed. The Bonferroni corrections were used to conduct post-hoc comparisons with the unexposed. We calculated odds ratios (*ORs*) with 95% confidence intervals (*CIs*) for risk of hyperuricemia by unconditional logistic regression, progressively adjusted for gender, marital status, famine severity, residence, smoking, drinking, BMI, hypertension, and diabetes. We assessed the multiplicative interaction of famine exposure, gender, residence, famine severity, BMI by the likelihood ratio test. A two-tailed *P* < 0.05 was considered as statistically significant. All statistical analyses were performed using Statistical Product and Service Solutions (SPSS, version 23.0).

## Results

Table 1 presents the characteristics of all participants according to the famine exposure in early life. The sample size of the unexposed, fetal-exposed, and early-childhood exposed subgroup were 1001, 574, 808, respectively. All three famine exposure subgroups resembled each other in terms of gender compositions (about 53–56% female), marital status (roughly 88–90% married and living with spouse), and residence (over 72% lived in rural). Additionally, more than 50% of all participants were overweight or obesity. The differences of smoking and hypertension between three famine exposure subgroups were statistically significant (*χ*^2^ = 6.345, *P* = 0.042; *χ*^2^ = 13.536, *P* < 0.001). Compared with the unexposed participants, the prevalence of smoking and hypertension for the early-childhood exposed was higher (*P* < 0.025). The prevalence of hyperuricemia among the unexposed, fetal-exposed, and early-childhood exposed subgroup was 10.7, 14.1, 11.1%, respectively.

**Table 1:** Characteristics of participants according to the Chinese famine exposure

	Unexposed	Fetal-exposed	Early-childhood exposed
*N* (%)	1001	574	808
Birthdate			
From Oct 1, year	1962	1959	1956
To Sep 30, year	1964	1961	1958
Gender, *n* (%)			
Male	441 (44.1)	256 (44.6)	380 (47.0)
Female	560 (55.9)	318 (55.4)	428 (53.0)
Marital, *n* (%)			
Married and living with spouse	902 (90.1)	508 (88.5)	713 (88.2)
Otherwise	99 (9.9)	66 (11.5)	95 (11.8)
Residence, *n* (%)			
Urban	274 (27.4)	142 (24.7)	208 (25.7)
Rural	727 (72.6)	432 (75.3)	600 (74.3)
Famine severity, *n* (%)			
Severely	631 (63.0)	338 (58.9)	493 (61.0)
Less severely	370 (37.0)	236 (41.1)	315 (39.0)
Smoking, *n* (%)	363 (36.3)	234 (40.8)	337 (41.7)†
Drinking, *n* (%)	378 (37.8)	218 (38.0)	292 (36.1)
Overweight/obesity, *n* (%)	541 (54.0)	292 (50.9)	410 (50.7)
Hypertension, *n* (%)	288 (28.8)	191 (33.3)	298 (36.9)‡
Diabetes, *n* (%)	135 (13.5)	92 (16.0)	136 (16.8)
Hyperuricemia, *n* (%)	107 (10.7)	81 (14.1)	90 (11.1)

†Between the groups, *χ*^2^ = 6.345, *P* = 0.042. Compared with the unexposed, *P* < 0.025 (Bonferroni correction)

‡Between the groups, *χ*^2^ = 13.536, *P* < 0.001. Compared with the unexposed, *P* < 0.025 (Bonferroni correction)

The associations between famine exposure and the risk of hyperuricemia are shown in Table 2. Compared with the unexposed participants, the unadjusted *OR* (95%*CI*) of hyperuricemia was 1.37 (1.01–1.87) for fetal-exposed participants. After adjustment for gender, marital status, famine severity, residence, smoking, drinking, BMI, hypertension, and diabetes, the *OR* of hyperuricemia for fetal-exposed participants was statistically significant (OR = 1.44; 95%*CI*: 1.05–1.98). Furthermore, even compared with unexposed and early-childhood exposed participants combined, an age-balanced control, and adjusted potential confounders, the famine exposure in the fetal stage was associated with an increased risk of hyperuricemia (Additional file 1: Table S2). However, consistent results were not observed in the early-childhood exposed participants, when compared with unexposed participants.

**Table 2:** Association of famine exposure in early life with hyperuricemia

	Unexposed	Fetal-exposed	Early-childhood exposed
Unadjusted			
*OR* (95% *CI*)	1.00	1.37 (1.01–1.87)	1.05 (0.78–1.41)
Adjusted			
*OR* (95% *CI*)	1.00	1.44 (1.05–1.98)	1.04 (0.77–1.42)

*Abbreviations*: *OR* odd ratio, *CI* confidence interval

As shown in Table 3, a multiplicative interaction was observed between famine exposure and BMI on hyperuricemia (*P*_interaction_ = 0.042). Compared with unexposed and BMI < 24 kg/m^2^ participants, the *OR* (95%*CI*) of hyperuricemia was 3.66 (2.13–6.30) for fetal-exposed and overweight/obesity participants after adjusted other covariates. When combined unexposed and early-childhood exposed participants as an age-balanced control, the interaction of famine exposure and BMI was not statistically significant (*P*_interaction_ = 0.054, Additional file 1: Table S3). Additionally, there was no statistical interaction between gender, famine severity, residence, and famine exposure on hyperuricemia (*P*_interaction_ > 0.05).

**Table 3:** Multivariable-adjusted *ORs* (95%*CI*) for the association of gender, BMI, and famine exposure in early life with hyperuricemia

	Unexposed	Fetal-exposed	Early-childhood exposed	*P*_interaction_
Gender^a^				0.374
Female	1.00	1.29 (0.80–2.06)	1.96 (1.17–3.28)	
Male	1.56 (0.95–2.58)	2.46 (1.43–4.26)	0.81 (0.50–1.30)	
Famine severity^b^				0.351
Less severity	1.00	1.31 (0.77–2.22)	1.27 (0.77–2.08)	
Severity	1.20 (0.78–1.85)	1.85 (1.16–2.96)	1.11 (0.70–1.76)	
Residence^c^				0.548
Rural	1.00	1.54 (1.05–2.25)	1.16 (0.81–1.67)	
Urban	1.29 (0.84–2.00)	1.61 (0.95–2.73)	1.03 (0.62–1.73)	
BMI, kg/m^2d^				0.042
< 24	1.00	2.58 (1.49–4.53)	1.58 (0.89–2.78)	
≥ 24	3.39 (2.06–5.57)	3.66 (2.13–6.30)	3.00 (1.78–5.06)	

*Abbreviations*: *OR* odds ratio, *CI* confidence interval, BMI body mass index

^a^ adjust marital status, famine severity, residence, smoking, drinking, BMI, hypertension, and diabetes

^b^ adjust gender, marital status, residence, smoking, drinking, BMI, hypertension, and diabetes

^c^ adjust gender, marital status, famine severity, smoking, drinking, BMI, hypertension, and diabetes

^d^ adjust gender, marital status, famine severity, residence, smoking, drinking, hypertension, and diabetes

## Discussion

In this study using data from the China Health and Retirement Longitudinal Survey, fetal famine exposure was associated with an increased risk of hyperuricemia in adulthood. The famine effect on hyperuricemia was accentuated by overweight/obesity. Compared with unexposed and BMI < 24 kg/m^2^ participants, those who were exposed to famine in the fetal stage and overweight/obesity had more than three times higher risk of hyperuricemia.

Previous studies have shown serum uric acid concentration would increase during starvation [15–17]. Furthermore, our study for the first time found that the experience of famine in the fetal stage was associated with hyperuricemia in later life. Accumulating evidence suggests that intrauterine malnutrition is associated with low fetal birth weight and low renal nephron numbers at all ages [28, 29]. The age-induced renal changes and structural changes may occur earlier in life because of intrauterine malnutrition, which means the occurrence of glomerular filtration rate decline is earlier [30]. The alterations in renal function may cause underexcretion of uric acid, which is likely to contribute to the development of hyperuricemia [16, 31, 32]. Further studies should be conducted to assess the effects of maladaptive adjustment to genes expression and renal dysfunction on hyperuricemia. In addition, the association between fetal famine exposure and hyperuricemia in adulthood did not change substantially after adjustment for overweight/obesity and other covariates, suggesting that overweight/obesity might not be a mediator for the association.

Our findings, consistent with previous studies [15, 33], indicated famine exposure in early life interacted with overweight/obesity during adulthood to influence hyperuricemia risk. Obesity has been linked to insulin resistance, which can facilitate the reabsorption of uric acid by augmenting the renal tubular sodium-hydrogen exchanger [34–36]. Additionally, impaired uric acid clearance is likely the main cause of hyperuricemia in obesity subjects because of the effect of hyperinsulinemia secondary to insulin resistance [34, 37]. As the thrifty phenotype hypothesis proposed, the occurrence of pathological changes following poor nutrition in early life may be dependent on the superimposition of risk factors in later life [38].

Consistent with some studies [20, 23, 39], we did not find any larger famine effects among adults born in severely affected versus less severely affected areas. This result may be explained by a great variation of famine severity in the county-level mortality [19], which caused the misclassification of famine severity. Further studies should be conducted that collect historical demographic records at the local level and find a more reliable indicator to better distinguish famine severity.

Additionally, we observed 52.5% of all participants aged 50–59 years in 2015 and 46.9% of men were overweight and obesity, which was higher than men aged 15–49 years (36.4%) in 2014 [40]. Thus, we might speculate whether age or famine effects could increase the prevalence of overweight and obesity. Although a previous study indicated the association between the risk of overweight, obesity and exposure to famine in early life in women aged 54–56 years but not in men [39], more detailed evidence and prospective cohorts are needed to confirm the risk of health caused by famine exposure in early life.

Several limitations of this study should be noted. First, individual famine exposure records were absent. The timing and severity of the Chinese famine varied greatly across regions. We used participant’s birthdate to distinguish famine exposure and computed excess mortality at the provincial level to measure famine severity. The misclassification bias was inevitable but should be nondifferential. To minimize potential misclassification, we excluded these participants born in the junction between three famine exposure subgroups. Second, medication information that may affect serum uric acid concentrations was not considered in the definition of hyperuricemia. Third, personal dietary information and family history of hyperuricemia was not available, which may be related to hyperuricemia. In addition, although the age-balanced control was used to avoid the age-related biases, the difference of age between the age-balanced control and fetal-exposed group still exists. Despite these limitations, this study has been among the first to investigate the long-term effects of early-life exposure to famine on hyperuricemia in Chinese adults.

## Conclusions

Famine exposure in the fetal stage was associated with an increased risk of hyperuricemia in adulthood. And the famine exposure effect on hyperuricemia was accentuated by overweight/obesity.

## Supplementary information

**Additional file 1: Table S1.** Distributions of missing data and outliners among three famine exposure subgroups. **Table S2.** Association of famine exposure in early life with hyperuricemia. **Table S3.** Multivariable-adjusted *ORs* (95%*CI*) for the association of BMI, and famine exposure in early life with hyperuricemia.

## Data Availability

The datasets are available in the website http://charls.pku.edu.cn/en

## Abbreviations

BMI: Body mass index
CHARLS: China Health and Retirement Longitudinal Study
CI: Confidence interval
HbA1c: Glycated hemoglobin
OR: Odds ratio
SUA: Serum uric acid
