# Supplementary material for: Early-life exposure to the Chinese famine of 1959–61 and risk of Hyperuricemia: results from the China health and retirement longitudinal study
Source: BMC Public Health. 2020 Jan 6;20:15. doi: 10.1186/s12889-019-8017-1 (PMC6945412; doi:10.1186/s12889-019-8017-1)
Supplement: Supplementary file 1 — Additional file 1: Table S1. Distributions of missing data and outliners among three famine exposure subgroups. Table S2. Association of famine exposure in early life with hyperuricemia. Table S3. Multivariable-adjusted ORs (95%CI) for the association of BMI, and famine exposure in early life with hyperuricemia. [file 12889_2019_8017_MOESM1_ESM.docx]

# Additional files

**Table S1** Distributions of missing data and outliners among three famine exposure subgroups

|  | Unexposed | Fetal-exposed | Early-childhood exposed |
| --- | --- | --- | --- |
| Missing |  |  |  |
| Uric acid | 3 | 1 | 0 |
| Weight/height | 18 | 11 | 13 |
| Blood pressure | 13 | 7 | 8 |
| Rural/urban | 1 | 3 | 5 |
| Smoking/drinking | 1 | 1 | 3 |
| Outliner |  |  |  |
| Weight/height | 3 | 3 | 5 |

**Table S2** Association of famine exposure in early life with hyperuricemia

|  | Unexposed and early-childhood exposed | Fetal-exposed |
| --- | --- | --- |
| Unadjusted |  |  |
| *OR* (95% *CI*) | Reference | 1.34 (1.02-1.78) |
| Adjusted |  |  |
| *OR* (95% *CI*) | Reference | 1.41 (1.06-1.88) |

*Abbreviations*: *OR* odd ratio, *CI* confidence interval

**Table S3** Multivariable-adjusted *ORs* (95%*CI*) for the association of BMI, and famine exposure in early life with hyperuricemia

|  | Unexposed and early-childhood exposed | Fetal-exposed | *P* _interaction_ |
| --- | --- | --- | --- |
| BMI, kg/m^2^ |  |  | 0.054 |
| < 24 | 1.00 | 2.03 (1.28-3.21) |  |
| ≥ 24 | 2.53 (1.80-3.56) | 2.88 (1.87-4.44) |  |

*Abbreviations*: *OR* odds ratio, *CI* confidence interval, BMI body mass index

Adjust gender, marital status, famine severity, residence, smoking, drinking, hypertension, and diabetes
